# Enhanced MHC Class‐II Expression in Fibroblastic Reticular Cells Associates with Maturation

**DOI:** 10.1002/eji.70086

**Published:** 2025-11-10

**Authors:** Janna E. G. Roet, Catarina Gago da Graça, Michael de Kok, Daphne Panocha, Tanja Konijn, Henk P. Roest, Luc J. W. van der Laan, Lisa G. M. van Baarsen, Charlotte M. de Winde, Reina E. Mebius

**Affiliations:** ^1^ Molecular Cell Biology & Immunology Amsterdam UMC Location Vrije Universiteit Amsterdam Amsterdam The Netherlands; ^2^ Amsterdam Institute for Immunology and Infectious Diseases Amsterdam The Netherlands; ^3^ Cancer Biology & Immunology Cancer Center Amsterdam Amsterdam The Netherlands; ^4^ Department of Surgery Erasmus MC Transplant Institute, University Medical Center Rotterdam Rotterdam The Netherlands; ^5^ Rheumatology & Clinical Immunology and Experimental Immunology Amsterdam UMC Location University of Amsterdam Amsterdam The Netherlands; ^6^ Amsterdam Rheumatology and Immunology Center (ARC) Amsterdam The Netherlands

**Keywords:** CD200, fibroblastic reticular cell, lymph node, MHC class II, single cell RNA sequencing

## Abstract

Autoimmunity can be initiated by autoreactive T cells that escaped central and peripheral tolerance induction. Peripheral tolerance in lymph nodes (LNs) is maintained by fibroblastic reticular cells (FRCs) via self‐antigen presentation in major histocompatibility complex (MHC) class II. FRCs can be divided into various subsets, with specific markers, functions, and locations. FRCs located in the T‐cell zone (TRCs) can express genes for antigen presentation in MHC class‐II, for example, *H2‐Ab1* and *Cd74*, as well as the immune inhibitory ligand *Cd200*. However, whether this can be linked to MHC class‐II protein expression and thus tolerance is unknown. By combining scRNAseq on murine FRCs with protein staining for extracellular MHC class‐II, we confirm that murine TRCs have the highest MHC class‐II transcript levels, while protein levels are elevated in multiple FRC subsets. Gene expression for MHC class‐II, as well as *Bst1* and *Cd200*, gradually increases along the pseudotime trajectory, with TRCs representing the end, indicating maturation. Finally, we validated in fresh LN cell suspensions that MHC class‐II protein expression is associated with murine BST1^+^ FRCs, independent of CD200, and with human BST1^+^CD200^+^ TRCs. This mature FRC subset, equipped to maintain peripheral tolerance, could be an interesting target for therapies against autoimmune diseases.

## Graphical abstract

Fibroblastic reticular cells (FRCs) in lymph nodes contribute to peripheral tolerance via MHC‐class‐II‐mediated antigen presentation. Using scRNAseq and protein analysis, we identify BST1^+^CD200^+^ FRCs, including the mature T cell‐zone FRCs, as key MHC‐class‐II expressing subsets in mice and humans, highlighting their potential role in immune regulation.

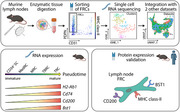

**Abbreviations**
apCAF: antigen‐presenting cancer‐associated fibroblasts
BSA: bovine serum albumin
BST1: bone marrow stromal cell antigen 1
CAF: cancer‐associated fibroblasts
FDC: follicular dendritic cell
FRC: fibroblastic reticular cell
IFRC: interfollicular reticular cell
LN: lymph node
MedRC: medullary reticular cell
MHC: major histocompatibility complex
MRC: marginal reticular cell
RT: room temperature
SC: stromal cell
scRNAseq: single‐cell RNA sequencing
TBRC: T‐B cell border reticular cell
TLS: tertiary lymphoid structure
TRC: T‐cell zone reticular cell
Treg: regulatory T cell
WT: wild type

## 1 Introduction

Autoimmunity is characterized by immune responses that are directed against healthy tissues and cells of an individual, which can cause severe tissue damage and autoimmune diseases, including rheumatoid arthritis and type 1 diabetes [1]. In a healthy situation, the tolerance of immune cells to recognize self‐antigens is controlled in both the thymus and secondary lymphoid organs. Central tolerance in the thymus is regulated by medullary thymic epithelial cells, which express and present self‐antigens in major histocompatibility complex (MHC) molecules to T cells. Recognition of presented self‐antigens by autoreactive T cells induces clonal deletion, or conversion into regulatory T cells (Tregs) [2, 3, 4]. However, some autoreactive T cells escape this mechanism and migrate into the periphery. Here, these cells are controlled by peripheral tolerance within secondary lymphoid organs, including lymph nodes (LNs) [5].

LNs have a dual role in immunity, as they facilitate a quick and effective immune response, while also providing the infrastructure for maintaining immune homeostasis and tolerance. Their compartmentalization in areas specific to B cells, T cells, and myeloid cells is crucial for optimal adaptive immune responses [6]. In addition, fibroblasts in the LN, known as fibroblastic reticular cells (FRCs), play a pivotal role in adaptive immunity by maintaining this LN integrity, coordinating cell trafficking, and controlling immune responses [6]. Moreover, FRCs can also express and (cross)present self‐antigens in MHC class‐I or MHC class‐II molecules, thereby interacting with autoreactive T cells [7, 8, 9, 10, 11, 12, 13, 14, 15, 16, 17]. In particular, it was shown using transgenic mice that FRCs derived from Keratine14 (K14)‐mOva mice present ovalbumin as a self‐antigen in MHC class‐II [16, 17]. Transplantation of K14‐mOva LNs in wild‐type (WT) mice, followed by injection of Rag2xOTII transgenic naive CD4^+^ T cells, which express ovalbumin‐specific T cell receptors, induced the conversion of CD4^+^ OTII T cells into Tregs. This process required the expression of MHC class‐II, IL‐2, and the co‐stimulatory molecules CD80/CD86. Subsequently, it was shown that these ovalbumin‐specific Tregs, when challenged with ovalbumin, suppressed the expansion of ovalbumin‐specific follicular T helper cells, as well as ovalbumin‐specific germinal center B cells [16, 17]. Similarly, human LN FRCs maintained Tregs, via IL‐2 and MHC class‐II expression, when co‐cultured with autologous CD4^+^ T cells [18]. Thus, FRCs contribute to peripheral tolerance and subsequent protection against autoimmune diseases.

FRCs form a heterogeneous cell population in the LN and, based on single‐cell transcriptomics, FRCs can be subtyped into different subsets, each with its own specific function and location [19, 20, 21, 22]. Of note, within the LN cortex, marginal reticular cells (MRCs) are located under the subcapsular sinus, at the surface of outer B‐cell follicles. Follicular dendritic cells (FDCs) occupy the primary B‐cell follicles and germinal centers, and interfollicular reticular cells (IFRCs) are present between the follicles. Within the paracortex, T–B cell border reticular cells (TBRCs) are enclosed by B‐cell follicles and T‐cell zones, where T‐cell zone reticular cells (TRCs) are situated. Medullary reticular cells (MedRCs) reside within the LN medulla, and pericytes surround the blood vessels [19, 20, 21, 22, 23].

As MHC class‐II protein expression by FRCs is necessary to maintain tolerance, it is of value to know how the expression is regulated, whether this is restricted to certain FRC subsets, and where these cells are located within the LN. It has been shown in mice that the Cxcl9‐expressing TRCs express several MHC class‐II genes (*H2‐Aa*, *H2‐Eb1*, and *H2‐Ab1*), as well as the invariant chain gene (*Cd74*) and peptide‐loading chaperone gene (*H2‐DMa*) [22]. While this is solely based on RNA levels, in this study, we investigate whether RNA expression can be linked to surface protein expression of MHC class‐II on murine FRCs and if MHC class‐II expression is induced upon differentiation, as described for the maturation of dendritic cells [24].

First, we demonstrate that the machinery for antigen presentation in MHC class‐II is restricted to fibroblasts present within the LNs, compared with other tissues. These LN fibroblasts are known as FRCs. Next, by combining scRNAseq with cell surface expression of MHC class‐II, we confirmed that TRCs have the highest gene expression of MHC class‐II compared with all other FRC subsets, while protein expression of MHC class‐II was elevated in multiple FRC subsets. Moreover, we show increased MHC class‐II gene expression along the pseudotime trajectory axis, in which the TRCs represent the end of the trajectory. We identified two surface expression markers, bone marrow stromal cell antigen 1 (BST1/CD157) and CD200, that followed a similar pseudotime pattern and were associated with the TRC subsets. Lastly, we validated in both mouse and human freshly digested LNs that MHC class‐II protein expression is associated with murine BST1^+^CD200^+/neg^ and human BST1^+^CD200^+^ TRCs. Altogether, we demonstrate that BST1^+^CD200^+/neg^ TRCs harbor the most MHC class‐II expressing FRCs, forming the mature subset that is equipped to maintain peripheral tolerance.

## 2 Results

### 2.1 Murine Fibroblasts Located in the Lymph Node Preferentially Express MHC Class‐II

**FIGURE 1:**
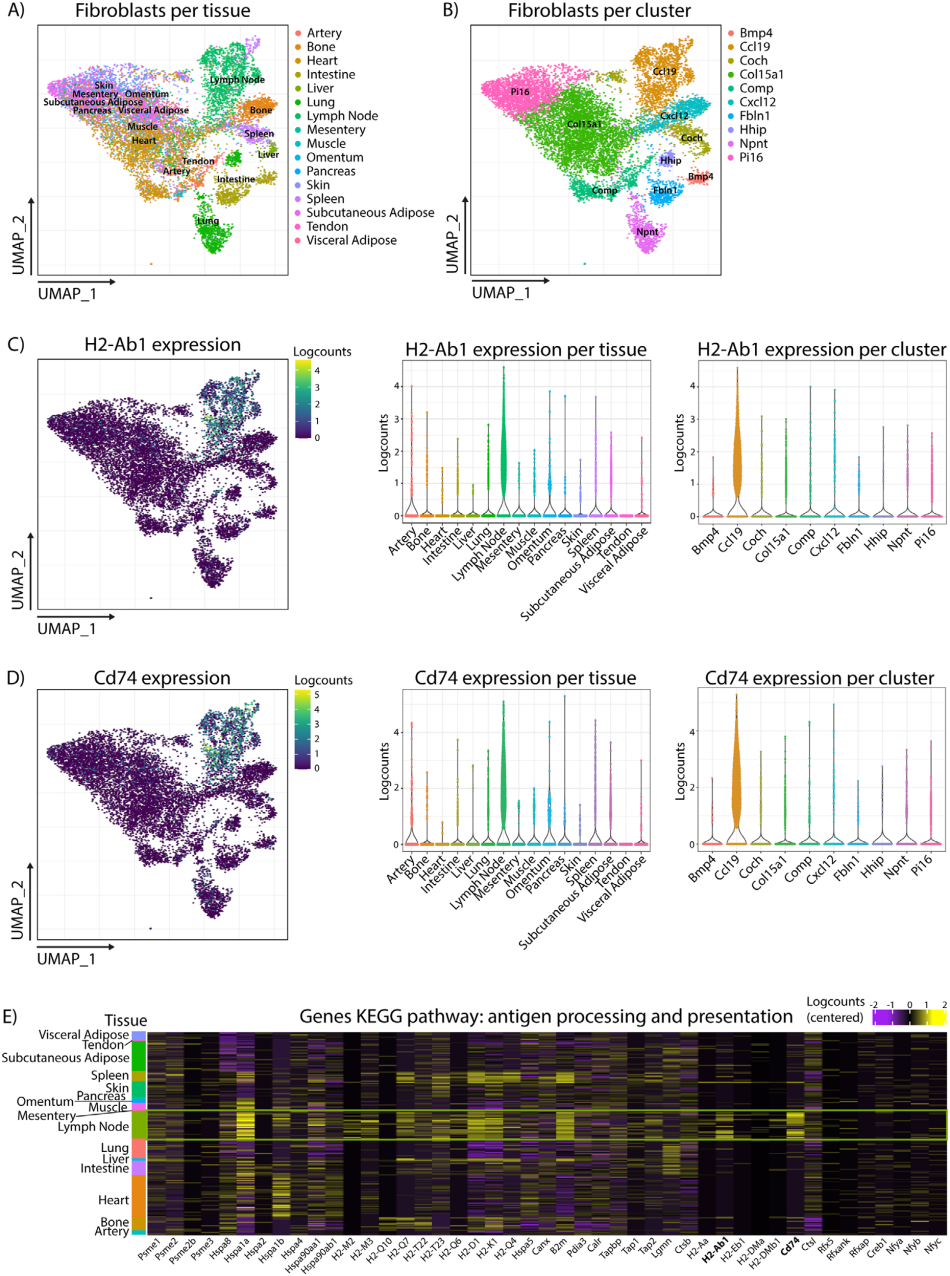
scRNAseq data of murine fibroblasts from various tissues during steady state. (A) UMAP visualization of the fibroblasts from 16 different tissues. (B) UMAP visualization of the fibroblasts per cluster. (C) *H2‐Ab1* gene expression visualized using UMAP dimensional reduction, violin plot per tissue, and violin plot per cluster. (D) *Cd74* gene expression visualized using UMAP dimensional reduction, violin plot per tissue, and violin plot per cluster. (E) Heatmap of the genes present in the KEGG pathway for antigen processing and presentation for the various tissues. All data are retrieved from Buechler et al. [25] and visualized using FibroXplorer.com.

The fibroblasts of the LN are known to play a pivotal role in immune regulation by presenting self‐antigens in MHC class‐II molecules [16, 17]. To identify whether fibroblasts in other organs could have the same functionality during homeostasis, we analyzed a publicly available single‐cell transcriptomics dataset, which contains data of murine fibroblasts from 16 different tissues during health and disease [25] (FibroXplorer.com). The fibroblasts of the LN are the main contributors to the Ccl19 cluster (Figure 1A,B). Interestingly, both *H2‐Ab1*, the gene for MHC class‐II, and *Cd74*, the invariant chain necessary for correct presentation of antigens in MHC class‐II, are preferentially expressed in fibroblasts located in the LN or belonging to the Ccl19 cluster (Figure 1C,D). Besides these two important genes for MHC class‐II expression, other genes belonging to the antigen processing and presentation pathway are also enriched for fibroblasts located in the LN (Figure 1E). Importantly, preferential expression of *H2‐Ab1* and *Cd74* is not found in fibroblasts from other lymphoid organs, like the spleen and omentum. When comparing the fibroblasts during various perturbed states, fibroblasts isolated from LNs during a viral infection are still the main contributors to the Ccl19 cluster and preferentially express *H2‐Ab1* and *Cd74*, as well as other antigen processing and presentation pathway genes, even when compared with fibroblasts isolated during and after lung viral infections, which are more closely matched inflammatory states (Figure S1). Overall, these data indicate that the machinery for antigen presentation in MHC class‐II is mainly restricted to fibroblasts within LNs, both during homeostasis and perturbations.

### 2.2 scRNAseq Reveals Five Main FRC Clusters

**FIGURE 2:**
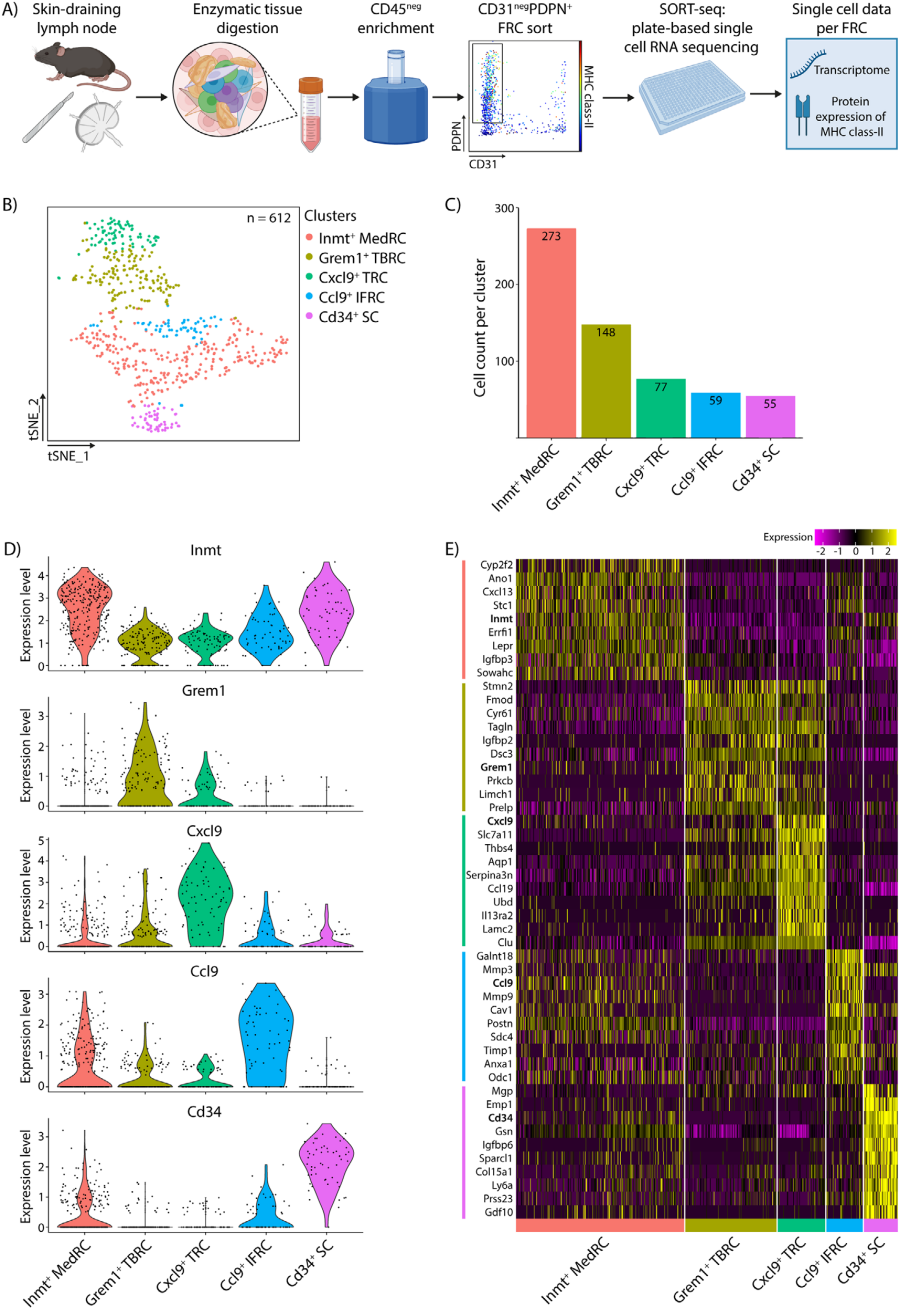
scRNAseq data of murine lymph node FRCs. (A) Schematic representation of the method used to isolate stromal cells from fresh murine skin‐draining lymph nodes and the next steps, including CD45^neg^ bead enrichment, sorting of CD31^neg^PDPN^+^ FRCs, and plate‐based single cell RNA sequencing, to obtain single cell data of the transcriptome and the protein expression of MHC class‐II per FRC. (B) Within the scRNAseq data, we identified five distinct clusters of FRC subsets, using unsupervised clustering and t‐SNE visualization. (C) Cell count per cluster identified in the transcriptome data. (D) Violin plots of gene expression per cluster of the main markers: *Inmt*, *Grem1, Cxcl9*, *Ccl9*, and *Cd34*. (E) Heatmap of the top 10 significantly differentially expressed genes with the highest log fold change per cluster. Created with BioRender.com.

Currently, multiple scRNAseq studies have shown that the fibroblasts of the LN, known as FRCs, can be divided into different subgroups, which can be characterized by the expression of different markers, including *Cd34*, *Ccl9*, *Cxcl9*, and *Grem1* [19, 20, 22]. While MHC class‐II molecules are described to be preferentially expressed in Cxcl9^+^ TRCs at the RNA level [22], it is important to determine whether MHC class‐II protein levels can be detected on particular subsets, since only those cells will have antigen presentation capacities in MHC class‐II. To evaluate this, we performed a combination of both protein staining for extracellular MHC class‐II, as well as scRNAseq on the same FRCs (*n* = 612) from murine skin draining LNs (Figure 2A). Performing unsupervised clustering on the scRNAseq data reveals five FRC clusters (Figure 2B,C). The clusters are annotated using cluster specific marker genes as reported earlier [19, 20, 22] (Figure 2D,E): Inmt^+^ MedRCs (*n* = 273), Grem1^+^ TBRCs (*n* = 148), Cxcl9^+^ TRCs (*n* = 77), Ccl9^+^ IFRC (*n* = 59), and Cd34^+^ stromal cells (SCs) (*n* = 55) (Figure 2B,D). By comparing our data with published datasets [19, 22], we could validate the overlap between cluster signatures (Figure S2).

The Inmt^+^ MedRCs express overlapping genes with the Inmt^+^ SC [19] and with both the Inmt^+^ SC and Nr4a1^+^ SC clusters [22] (Figure S2). These clusters populate the medullary cords and are described as MedRCs [20, 22, 26]. Interestingly, some genes belonging to MRCs, including *Cxcl13*, are expressed within the Inmt^+^ MedRC cluster, suggesting that this cluster also contains some MRCs in our dataset.

The Grem1^+^ TBRCs show overlapping genes with the Ccl19^low^ clusters of both Kapoor et al. [19] and Rodda et al. [22]. Grem1^+^ FRCs are localized at the interface of B‐ and T‐cell zones and have been described as TBRCs.

The Cxcl9^+^ TRCs express overlapping genes with both the Ccl19^high^ TRCs and Cxcl9^+^ TRCs from Kapoor et al. [19] and Rodda et al. [22]. Both clusters have been described as being located within the T‐cell zone and annotated as TRC [19, 22].

The Ccl9^+^ IFRCs show overlapping genes with the Cd266^+^ SCs [19]. The FRCs within the interfollicular regions showed the highest *Ccl9* expression, which led us to annotate these cells as IFRCs [20].

Lastly, the Cd34^+^ SCs express overlapping genes with all four Cd34^+^ SC clusters of Kapoor et al. [19] and with the Cd34^+^ cluster of Rodda et al. [22]. However, we do not have the resolution in our dataset to distinguish multiple Cd34^+^ SC clusters. Moreover, we could not distinguish separate clusters for pericytes or FDCs. Overall, our scRNAseq dataset contains five main FRC clusters that overlap with existing literature.

### 2.3 Elevated MHC Class‐II mRNA and Protein Expression in TRCs

**FIGURE 3:**
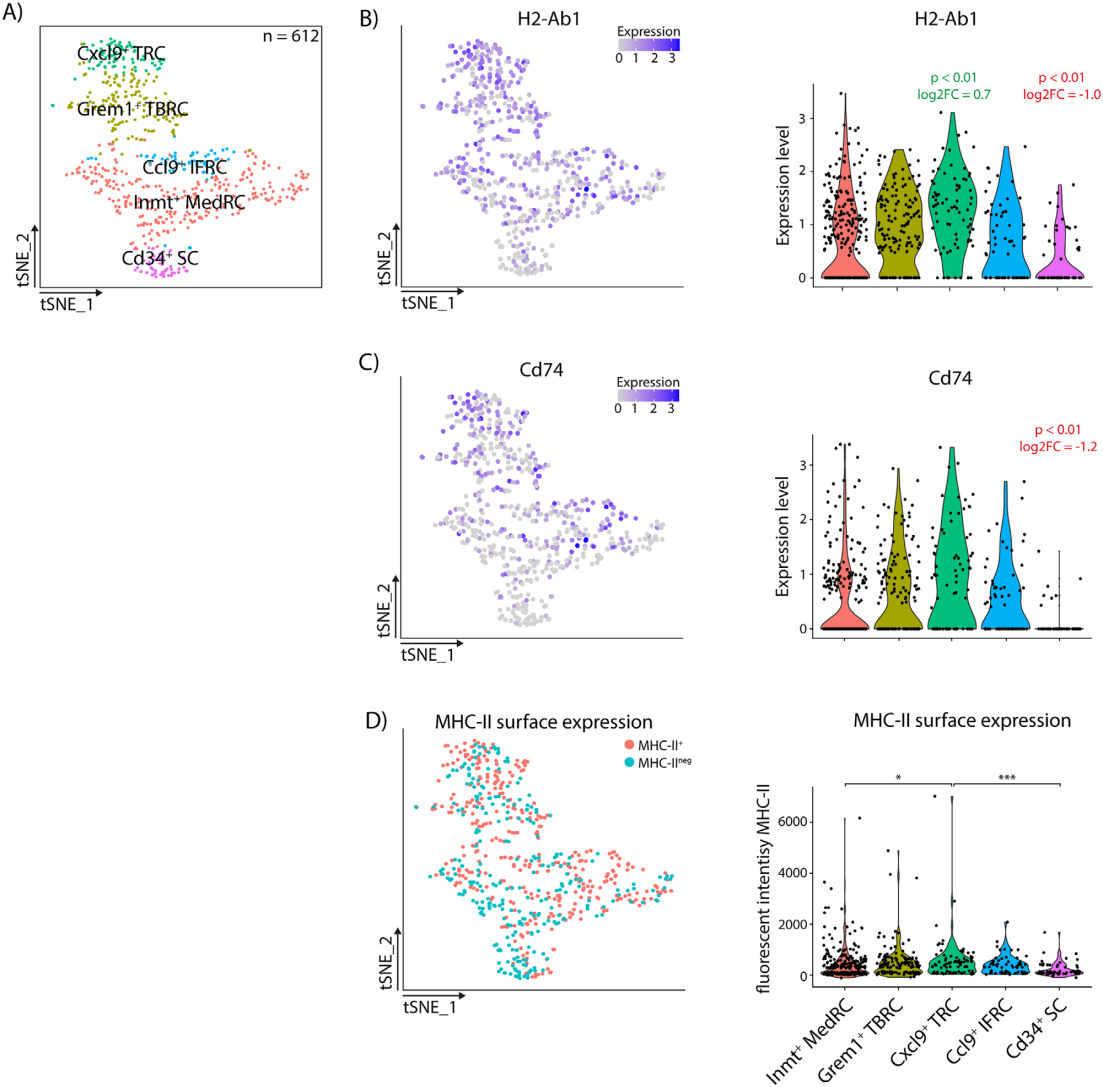
Gene and protein expression of MHC class‐II on murine lymph node FRCs. (A) t‐SNE visualization of the five FRC clusters in the transcriptome data. (B) *H2‐Ab1* gene expression visualized using t‐NSE dimensional reduction and violin plot per cluster. *H2‐Ab1* is differentially expressed in Cxcl9^+^ TRC with a log2FC of 0.7 and in Cd34^+^ SC with a log2FC of −1.0, compared with all other clusters. (C) *Cd74* gene expression visualized using t‐SNE dimensional reduction and violin plot per cluster. *Cd74* is differentially expressed in Cd34^+^ SC with a log2FC of ‐1.2, compared with all other clusters. D) MHC class‐II protein surface expression visualized as MHC class‐II^neg^ or MHC class‐II^+^ using t‐SNE dimensional reduction and as fluorescent intensity of MHC class‐II using a violin plot per cluster. Kruskal–Wallis test with Dunn's multiple comparisons test, *p < 0.05, ***p < 0.001. Log2FC = log 2‐fold change, identified using the “FindAllMarkers” function.

Within the five FRC clusters (Figure 3A), mRNA levels for *H2‐Ab1* are not restricted to one cluster (Figure 3B). Interestingly, *H2‐Ab1* is differentially higher expressed in Cxcl9^+^ TRCs (positive fold change) and lower expressed in Cd34^+^ SCs (negative fold change), compared with the other clusters. Moreover, all clusters also contained mRNA levels for *Cd74* (Figure 3C), while *Cd74* is only differentially lower expressed in Cd34^+^ SCs (negative fold change), compared with the other clusters. This suggests that on the mRNA level, all FRC clusters, except for Cd34^+^ SCs, have the potential to express the proteins necessary for antigen presentation via MHC class‐II, with the Cxcl9^+^ TRCs having elevated levels of *H2‐Ab1*, as described earlier for TRCs [22]. A similar pattern is seen for the extracellular protein expression of MHC class‐II on the same FRCs, as all clusters contain cells that express MHC class‐II (Figure 3D), including the Cd34^+^ SCs. This difference between MHC class‐II transcript and protein expression in the Cd34^+^ SCs might result from the capture of MHC class‐II protein from dendritic cells [7], explaining protein expression while lacking transcripts for MHC class‐II genes. The Cxcl9^+^ TRCs have significantly higher MHC class‐II protein expression compared with Inmt1^+^ MedRCs and Cd34^+^ SCs. However, no significant difference in MHC class‐II protein expression is observed between Cxcl9^+^ TRCs and Grem1^+^ TBRCs or Ccl9^+^ IFRCs. To conclude, the Cxcl9^+^ TRCs have the highest transcript levels of *H2‐Ab1* compared with all other clusters, while the MHC class‐II protein expression in these cells is similar to Grem1^+^ TBRCs and Ccl9^+^ IFRCs, but significantly higher than Inmt^+^ MedRCs and CD34^+^ SCs.

### 2.4 MHC Class‐II mRNA Expression Increases along the Pseudotime Trajectory of FRCs

**FIGURE 4:**
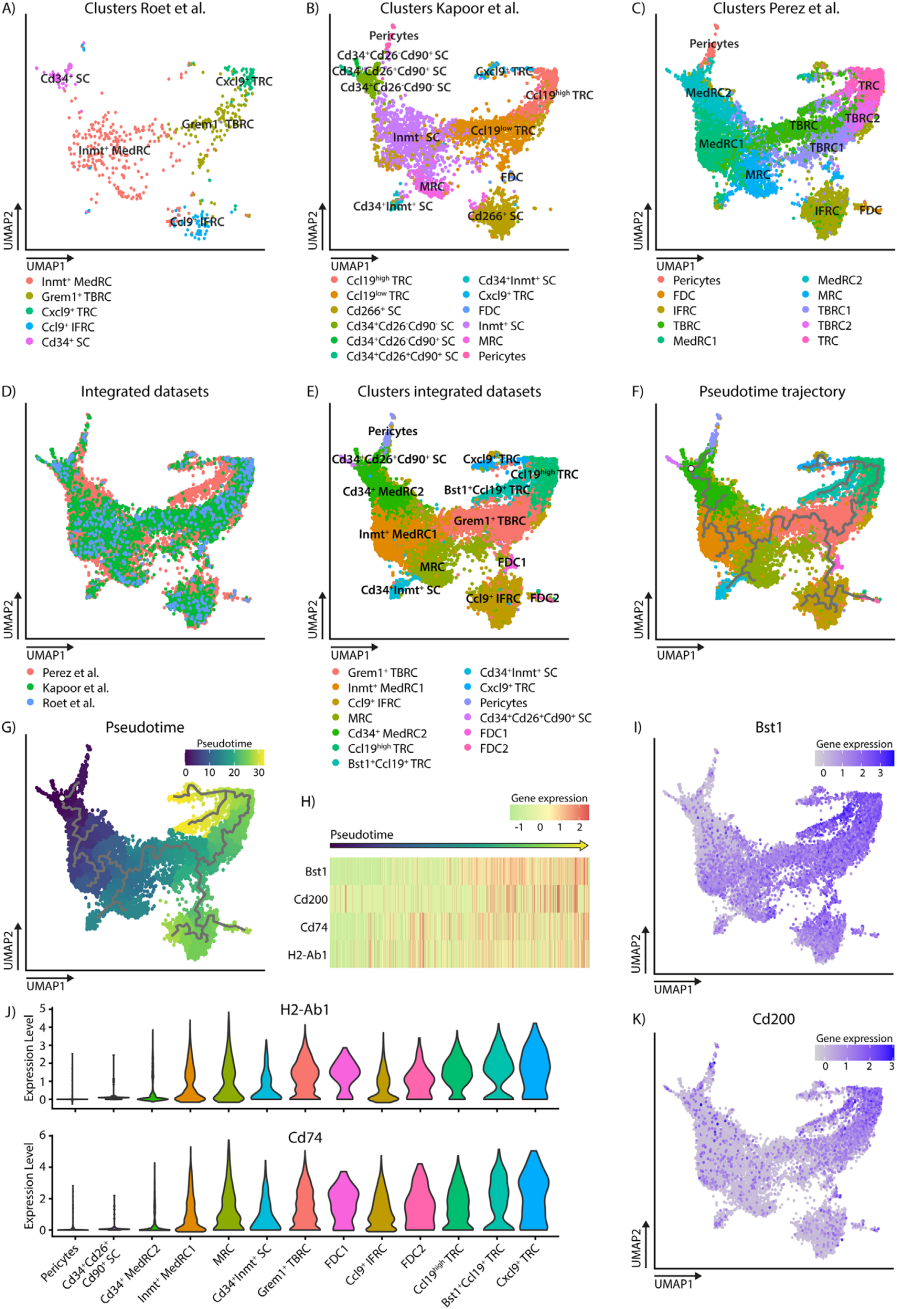
TRCs represent the end of the FRC pseudotime trajectory, together with Bst1 and Cd200 expression. UMAP visualization of the previously annotated clusters of the integrated murine lymph node FRC scRNAseq datasets from (A) Roet et al. (2025) (this manuscript), (B) Kapoor et al. [19], and (C) Perez‐Shibayama et al. [20]. (D) UMAP visualization of the overlap of the three integrated datasets. (E) Unsupervised clustering of the integrated dataset identified 13 FRC clusters. UMAP visualization of pseudotime analysis on the integrated dataset, as shown in (F) trajectory or (G) pseudotime. The white circle represents the starting point. (H) Heatmap of genes expressed along the pseudotime axis. UMAP visualization of gene expression of (I) *Bst1* and (K) *Cd200* in the integrated dataset. (J) Violin plot of *H2‐Ab1* and *Cd74* gene expression per cluster, ordered on pseudotime.

To enlarge our scRNAseq dataset, we next integrated our dataset with two comparable publicly available datasets of murine FRCs [19, 20]. The annotation for each cluster in both datasets has been described, allowing for comparison of the cluster nomenclature for each dataset separately (Figure 4). For the dataset of Kapoor et al. [19], the clusters with annotation was publicly available (Figure 4B), and for the dataset of Perez‐Shibayama et al. [20], we performed cluster analysis, removed two endothelial clusters and two clusters highly expressing mitochondrial genes, indicating apoptotic or lysing cells, after which we annotated the clusters based on the markers described before for the different clusters (Figure 4C) [20]. Interestingly, this analysis led to the clustering of three TBRC clusters expressing *Grem1* and *Tnfsf13b*, in contrast to the two populations described before [20].

The dimensionality reduction of the combined dataset shows overlap of all three datasets (Figure 4D). By comparing the annotation of the clusters within the different datasets, we could validate the annotation of our scRNAseq dataset, as similar clusters overlap. Next, unsupervised clustering on the integrated scRNAseq data revealed 13 FRC clusters (Figure 4E). Combining the information of the annotated clusters from the three original datasets, we could annotate the integrated clusters as: Grem1^+^ TBRC, expressing *Grem1* and *Tnfsf13b*; Inmt^+^ MedRC1, expressing *Inmt*, *Nr4A1, Cxcl1, Cxcl2* and *Ccl7*; Ccl9^+^ IFRC, expressing *Ccl9*; MRC, expressing *Hamp2* and *Tnfsf11*; Cd34^+^ MedRC2, expressing *Inmt*, *Nr4a1, Dpep1* and some *Cd34*; Ccl19^high^ TRC, expressing *Ccl19* and *Bst1*; Bst1^+^Ccl19^+^ TRC, expressing *Ccl19* and *Bst1*; Cd34^+^Inmt^+^ SC, expressing *Cd34, Inmt* and *Col3a1*; Cxcl9^+^ TRC, expressing *Cxcl9*; Pericytes, expressing *Mcam*, *Itga7*, and *Acta2*; Cd34^+^Cd26^+^Cd90^+^ SC, expressing *Cd34* and *Pi16*; FDC1 and FDC2, expressing *Cr2* and *Cxcl13* (Figure 4E; Figure S3). Some of these subsets have been described for their potential role in tolerance and inflammation. The Cxcl9^+^ TRCs might be an activated subset of the Ccl19^high^ TRCs that can tolerize T cells in a resting LN via expression of MHC class‐II [19, 22, 27], while the Grem1^+^ TBRCs engage with DCs and are pivotal in primary T cell responses [19]. To identify whether the expression of MHC class‐II on FRCs is increased upon differentiation, as seen for example on the surface of dendritic cells during maturation [24], we performed pseudotime trajectory analysis on the integrated dataset (Figure 4F,G). The starting point of the trajectory was selected near the Cd34^+^Cd26^+^Cd90^+^ SCs, as these cells were described before as the start of the FRC trajectory [19, 28]. From here, the pseudotime lineage displays a gradual transition of FRCs through MedRCs, splitting into MRCs to Cd34^+^Inmt^+^ SCs and TBRCs. From the TBRCs, the pseudotime trajectory branches into B‐cell area FRCs, via FDC1, Ccl9^+^ IFRCs into FDC2, and T‐cell area FRCs, via Ccl19^high^ TRCs into both Bst1^+^Ccl19^+^ TRCs and Cxcl9^+^ TRCs at the end of the pseudotime trajectory (Figure 4F,G). Both *Cd74* and *H2‐Ab1* expression on FRCs seems to gradually increase along the pseudotime axis (Figure 4H), which is reflected in the expression of *Cd74* and *H2‐Ab1* within the clusters when ordered according to their localization on the pseudotime axis (Figure 4J). We identified two surface expression markers that followed a similar pseudotime pattern and that were associated with the TRC subsets, located at the end of the pseudotime trajectory: *Bst1* and *Cd200* (Figure 4H). *Bst1* is broadly expressed across FRC clusters, except for Cd34^+^ MedRC2, Cd34^+^Cd26^+^Cd90^+^ SCs and pericytes, and the highest expression is present in the Ccl19^high^ TRCs and Bst1^+^ Ccl19^+^ TRCs (Figure 4I). Similarly, *Cd200* also showed the highest expression in the Ccl19^high^ TRCs and Bst1^+^Ccl19^+^ TRCs (Figure 4K). Overall, using the integrated murine FRC scRNAseq dataset, we could identify 13 FRC clusters. The pseudotime analysis shows an FRC trajectory from Cd34^+^ FRCs, via MedRCs and TBRCs, into B‐cell area FRCs and T‐cell area FRCs, with a gradual increase of *Cd74*, *H2‐Ab1*, *Bst1*, and *Cd200* expression along this pseudotime trajectory. Thus, suggesting that MHC class‐II expression is a trait of more differentiated TRCs.

### 2.5 Enhanced MHC Class‐II Protein Expression on Murine BST1^+^CD200^+/neg^ FRCs and Human BST1^+^CD200^+^ TRCs

**FIGURE 5:**
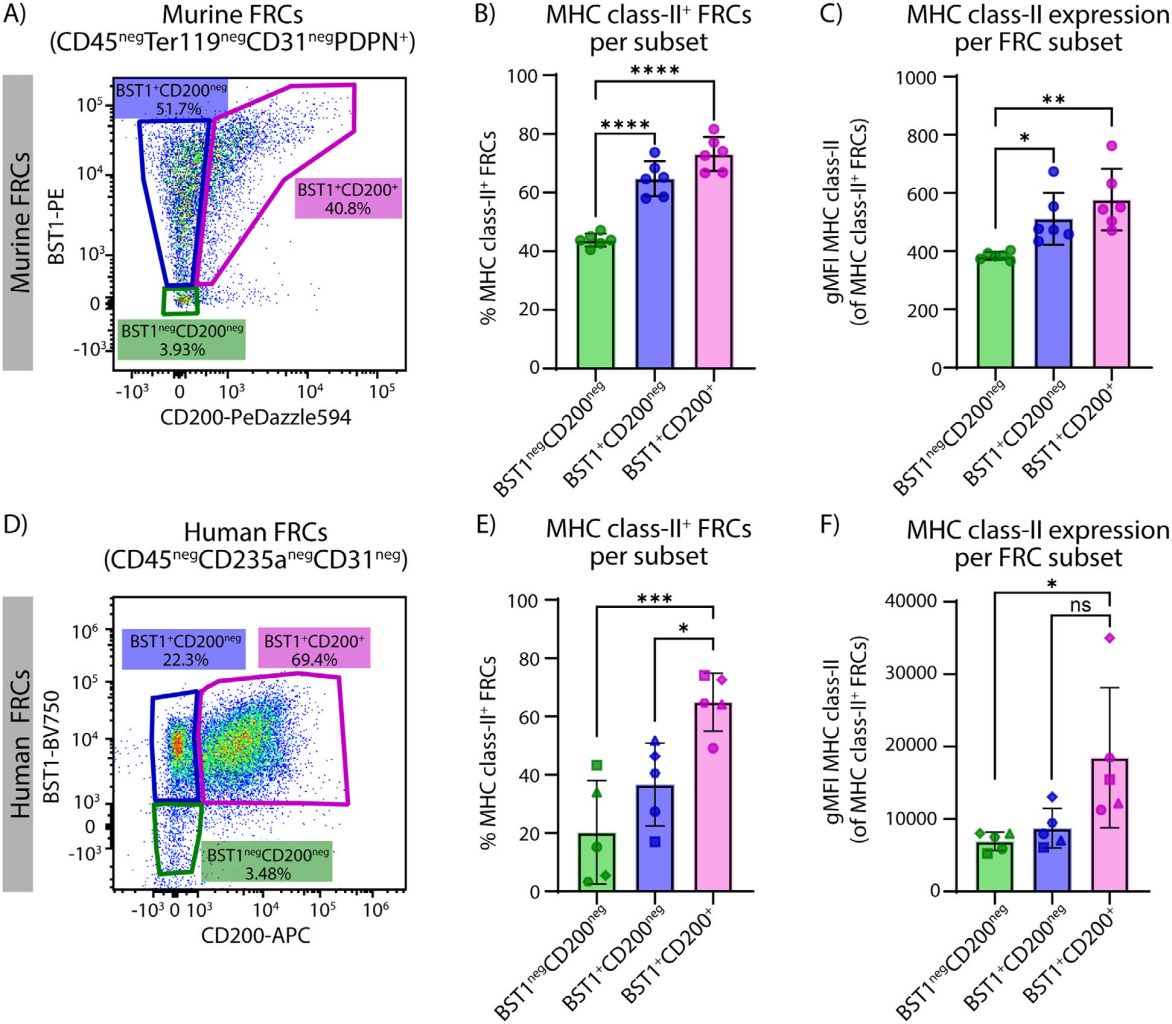
Highest MHC class‐II protein expression on BST1^+^ and CD200^+^ murine and human FRCs. (A) Flow cytometry data showing the gating strategy based on CD200 and BST1 expression of freshly digested murine lymph node FRCs (CD45^neg^CD31^neg^PDPN^+^). (B) Percentage of MHC class‐II^+^ murine FRCs (*n* = 6). (C) gMFI of cell surface protein expression of MHC class‐II on MHC class‐II^+^ murine FRCs (*n* = 6). (D) Flow cytometry data showing the gating strategy based on CD200 and BST1 expression of freshly digested human lymph node FRCs (CD45^neg^CD235a^neg^CD31^neg^). (E) Percentage of MHC class‐II^+^ human FRCs (*n* = 5). (F) gMFI of cell surface protein expression of MHC class‐II on MHC class‐II^+^ human FRCs (*n* = 5). Data are represented as mean ± standard deviation. One‐way ANOVA with a Tukey's multiple comparison test, *p < 0.05, **p < 0.01, ***p < 0.001, ****p < 0.0001, ns: not significant, gMFI: geometric mean fluorescent intensity.

BST1 can be expressed on many cell types, including myeloid, endothelial, mesothelial, mesenchymal stem cells, and FRCs, especially on TRCs [19, 29]. In general, BST1 can bind to the extracellular matrix, affecting cell adhesion, growth, survival, migration, and proliferation [29]. CD200 can also be present on various cell types, including myeloid, lymphoid, epithelial, and cancer cells, as well as on the specific FRC subsets TRCs and FDCs [19, 30, 31]. CD200 is the ligand for the immune inhibitory receptor CD200R, which is expressed on myeloid, T, and B cells [32]. Using CD200 and BST1 cell surface protein expression during flow cytometry of freshly digested murine LNs, we could distinguish BST1^neg^CD200^neg^ FRCs (3.9%), BST1^+^CD200^neg^ FRCs (51.7%), and BST1^+^CD200^+^ FRCs (40.8%), after pregating on FRCs (CD45^neg^Ter119^neg^PDPN^+^CD31^neg^) (Figure 5A; Figure S4A). Similar to the mRNA expression, most FRCs express BST1, of which some also express CD200. Interestingly, compared with the BST1^neg^CD200^neg^ FRCs, a significantly higher percentage of both BST1^+^CD200^neg^ and BST1^+^CD200^+^ FRCs express MHC class‐II, while also expressing more MHC class‐II as indicated by increased fluorescence intensity (Figure 5B,C). Overall, in mice, the MHC class‐II protein expression is enhanced in BST1^+^ FRCs, independent of CD200 expression, and, as *Bst1* expression is broadly expressed across FRC clusters (Figure 4I), this is not restricted to a specific FRC subset.

To investigate whether this could be translated to the human setting, we performed a similar protein staining on freshly digested healthy human LNs. In here, after pregating on FRCs (CD45^neg^CD235a^neg^CD31^neg^) [33], we could distinguish BST1^neg^CD200^neg^ FRCs (3.8%), BST1^+^CD200^neg^ FRCs (23.1%), and BST1^+^CD200^+^ FRCs (68.8%) (Figure 5D; Figure S4C). In contrast to the murine setting, only the BST1^+^CD200^+^ FRCs have a significantly higher percentage of MHC class‐II^+^ FRCs compared with both the BST1^neg^CD200^neg^ and BST1^+^CD200^neg^ FRC subsets (Figure 5E). Moreover, within the MHC class‐II^+^ FRCs, the expression of MHC class‐II is significantly higher on the BST1^+^CD200^+^ FRCs compared with the BST1^neg^CD200^neg^ FRCs (Figure 5F). This suggests that CD200 expression associates with MHC class‐II expression in human FRCs. CD200 expression has been described for both murine FRCs located within the T cell zone [19], as well as for murine and human FDCs in the B cell follicle [22, 31, 34]. We could confirm this CD200 expression in both the T cell zone and the B cell follicle within human LNs (Figure S5). Of note, while BST1, CD200, and MHC class‐II expression have all been described for FDCs as well [22, 31, 34], only between 1–9% per subset consisted of CD21^+^ FDCs (Figure S4E,F), indicating that still most of these cells are other subsets of FRCs. Moreover, CD200 expression has also been described for murine FRCs located within the T cell zone [19], which we could confirm for human LNs (Figure S5), suggesting that the BST1^+^CD200^+^ FRCs are TRCs. Thus, MHC class‐II protein expression is associated with murine BST1^+^CD200^+/neg^ FRCs and human BST1^+^CD200^+^ TRCs.

## 3 Discussion

Peripheral tolerance in LNs is maintained by LN stromal cells via presentation of self‐antigens in MHC class‐II to autoreactive T cells [16, 17]. Here, we uncovered that fibroblasts in the LN have the highest expression of MHC class‐II‐associated genes compared with other tissues, and we identified the subset of LN fibroblasts that has the highest expression of MHC class‐II, at both RNA and protein levels: BST1^+^CD200^+/neg^ murine FRCs and BST1^+^CD200^+^ human FRCs, known to locate in the T‐cell zone [19]. The expression of the genes involved in antigen presentation increases along the pseudotime differentiation axis, with the TRC subsets representing the end of the trajectory. This suggests that the peripheral tolerance induced by FRCs is regulated by the more differentiated TRCs, located in the T‐cell zone, where there is an optimal probability for T cell‐FRC interactions.

Our findings are in line with earlier reports, where BST1 has been described as a classical marker for T‐cell zone FRCs [19], while also being expressed by MRCs and FDCs, but not by MedRCs [22, 26]. This is consistent with the expression pattern identified for *Bst1* in the integrated FRC dataset (Figure 4I). On the contrary, CD200 is not a commonly used marker for FRCs, although it has been described by Kapoor et al. [19] that *Cd200* is mostly expressed on the Ccl19^high^ TRC subset and that *Cd200* expression identified a subset of Grem1^+^ TRCs, localized deeper within the T‐cell zone in mice [19]. Moreover, CD200 expression has also been identified on FDCs, localized within the B‐cell follicle [31]. Interestingly, within rheumatoid arthritis, a chronic inflammatory autoimmune disorder, pro‐resolving CD200^+^ fibroblasts arise during the resolution of inflammation in the synovium. These CD200^+^ fibroblasts have the capacity to dampen inflammatory pathways, as identified with gene‐set enrichment analysis, and facilitate arthritis resolution via CD200‐CD200R1 signaling on type 2 innate lymphoid cells [35]. In murine nose‐draining LNs, it has been shown that CD200 expression is essential for the induction of intranasal tolerance and that Tregs cannot be induced in the absence of CD200^+^ cells. This study further showed that both LN stromal cells, as well as activated T cells, could provide CD200 to CD200R‐expressing antigen‐presenting DCs, necessary to induce tolerance [36]. During homeostasis, CD200 is only expressed on LN stromal cells, which could therefore be instrumental in homeostatic conversion to or maintenance of Tregs [36]. Overall, CD200 expression on fibroblasts has been described to dampen inflammatory pathways or promote tolerance via CD200‐CD200R signaling. While we found on human FRCs that the BST1^+^CD200^+^ TRCs have the highest expression of MHC class‐II, suggesting that this subset of FRCs plays a role in peripheral tolerance, the immune inhibitory function of CD200 on FRCs needs to be further explored, for example, by using CRISPR‐Cas9 knock‐outs of CD200 in human LN FRCs.

We identified that specifically fibroblasts from the LN express genes associated with antigen presentation and tolerance induction, including MHC class‐II, compared with fibroblasts from various other tissues, such as the lung. Could this be due to the unique expression of known regulators of MHC class‐II within the LN fibroblasts? It has been shown that MHC class‐II expression on FRCs is regulated by the class‐II transactivator (CIITA), exclusively via the expression of promoter IV (pIV) of CIITA [7, 27]. Unfortunately, we could not compare the expression of these regulators between the fibroblasts from various tissues, as they were not present within this dataset. Future research should elucidate the molecular pathway of MHC class‐II regulation in fibroblasts in LNs and across tissues.

Would every fibroblast be able to express MHC class‐II to induce tolerance, as long as it has enough interactions with immune cells, or is this function imprinted early during ontogeny in FRCs? It has been shown that postnatal tissue‐resident fibroblasts can retain their plasticity and can alter their functional phenotype to form tertiary lymphoid structures (TLSs) [37, 38, 39]. However, it is unknown whether these TLS fibroblasts express MHC class‐II and can have tolerogenic functions. Currently, it is believed that TLSs amplify immune responses, as the presence of TLSs in chronic autoimmune conditions has been associated with poor prognosis, while in cancer, it has been considered a favorable prognostic marker, contributing to antitumor immune responses [37, 38, 40]. Moreover, autoreactive cells were shown to proliferate and survive within TLSs, suggesting that fibroblasts within TLSs are not able to repress autoreactive cells [40]. Interestingly, based on gene expression and chromatin accessibility, it was shown that fibroblasts of nonlymphoid organs have an epigenetically encoded immune potential, while a tolerance potential was not mentioned [41]. Overall, although tissue‐resident fibroblasts can alter their phenotype to form TLSs and support immune interactions as seen in LNs, they seem to only induce immune activation and not immune tolerance.

Within the context of tumors, a subtype of cancer‐associated fibroblasts (CAFs) can express diverse MHC class‐II molecules and are identified as antigen‐presenting CAFs (apCAFs) [42]. CAFs can be derived from tissue‐resident fibroblasts, as well as epithelial cells, endothelial cells, pericytes, adipocytes, smooth muscle cells, or mesenchymal stem cells [43]. By investigating the transcriptome of nine solid tumor types, apCAFs were identified in all nine tumor types and were mainly derived from tissue‐resident fibroblasts [44]. However, these apCAFs were associated with immune activation and antitumor effects [44]. Interestingly, another study identified that apCAFs in pancreatic cancer induced Treg formation, suggesting an immunosuppressive role, but these apCAFs were derived from mesothelial cells and not tissue‐resident fibroblasts [45]. Together, these observations suggest that the tolerogenic function as seen for FRCs is not a common feature of tissue‐resident fibroblasts upon immune cell interactions or differentiation into CAFs, and might be imprinted early during the ontogeny of FRCs.

The understanding of the role of FRCs within the LN is mostly based on murine studies [16, 17, 20, 21, 22, 28, 34, 46]. In recent years, more human‐based studies have been performed to translate these findings to a human setting [19, 26, 31, 33, 47, 48]. However, differences between murine and human FRCs have been indicated. For example, PDPN is historically used as a hallmark marker for FRCs, as it is broadly expressed on mesenchymal stromal cells within mouse LNs. However, within human LNs, a large portion of freshly isolated mesenchymal stromal cells lack PDPN expression [33, 47, 48], and human PDPN^neg^ mesenchymal LN stromal cells can have similar characteristics to murine PDPN^+^ FRCs [47, 48]. Therefore, it was suggested to define human FRCs as CD45^neg^CD31^neg^ [33], which was also used for the gating of human FRCs compared with CD45^neg^CD31^neg^PDPN^+^ gating for murine FRCs in this study.

Besides the differences in marker expression, functional differences between mouse and human FRCs have also been described. In mouse LNs, the FRCs regulate T‐cell proliferation via their production of nitric oxide [46, 49, 50]. On the contrary, human FRCs utilize four other pathways, all independent of nitric oxide, for the control of T‐cell proliferation as well as differentiation [48]. Within this current study, we observed a difference in MHC class‐II expression in different FRC subsets between mouse and human FRCs: the cells with the highest expression of MHC class‐II were present in either all BST1^+^ murine FRCs, independent of CD200 expression, or specifically in BST1^+^CD200^+^ human FRCs. It is unclear whether these are biological differences between species or due to the differences in immunological history between human and mice: while most murine studies are based on immunologically naive mice, which have never encountered an infection, the human LNs that are studied will have undergone various infections during a person's lifespan. Moreover, these differences could also be affected by the different relative ages of the mice and human donors. The protein expression levels of MHC class‐II on the different FRC subsets were determined in mature adult 6‐month‐old mice. The human equivalent of this life phase would range from 20 to 30 years old [51]. However, four out of the five human donors analyzed were in a later life phase, between the ages of 46 and 81 years. Age‐associated changes have been shown for murine FRCs, including impaired differentiation of FRCs and increased fibrosis [52], suggesting that the observed differences might also be affected by the different relative ages of the mice and human donors. It would be interesting to identify whether either older mice and/or several rounds of self‐limiting infections affect the murine FRCs and might be a more representative model for human FRCs.

In conclusion, we uncovered the subset of FRCs during homeostasis that expresses the highest levels of MHC class‐II, necessary for peripheral tolerance. It would be interesting to investigate what happens to these cells in a reactive LN. FRCs have a dual role in the control of adaptive immune responses: they can both enhance immune activation and dampen excessive inflammation [6]. However, it remains unknown whether these different roles are divided over different FRC subsets or whether FRC subsets have an overlap in functionality. Interestingly, extended proinflammatory responses have been associated with the onset of autoimmune diseases [53, 54, 55], as well as a changed LN stromal environment, as seen during the early phases of rheumatoid arthritis [56, 57, 58]. This suggests that a potential malfunctioning of FRCs to dampen inflammation and maintain tolerance makes space for autoreactive immune cells to develop. This indicates that, especially the FRC subset important to maintain tolerance, could be an interesting target for therapy.

## 4 Data Limitations and Perspectives

We Acknowledge That the Current Study Is Subject to Certain Limitations. This includes low cell number (*n* = 612) for the combined FRC scRNAseq and protein expression dataset, resulting in identifying fewer clusters as compared with other public FRC datasets [19, 20, 22]. Moreover, we sorted specifically CD31^neg^PDPN^+^ FRCs, as PDPN expression has been shown to be a key transcriptional regulator of FRC function in murine LNs [59], while other FRC datasets applied less stringent filters, including, for example, all nonendothelial stromal cells [19, 22] or all Ccl19^+^ stromal cells [20]. This could have further reduced the presence of shared subsets across the datasets. However, by comparing the clusters identified in this study with the clusters from other datasets, we could assign all our clusters to one or more of the other clusters (Figure S2), suggesting a full coverage of the FRCs present in the LN. To enhance the cell number and thereby the power to perform pseudotime analysis, we integrated our scRNAseq dataset with two publicly available murine FRC scRNAseq datasets [19, 20]. All cells were harvested from skin‐draining LNs, from either 6 weeks (this manuscript), 5–9 weeks [19], or 8–10 weeks [20] old naive mice. The cells used for sequencing were CD45^neg^CD31^neg^PDPN^+^ sorted cells (this manuscript), CD45‐depleted LN cells of which the mesenchymal stromal cells (CD45^neg^CD31^neg^PDPN^+/neg^) were used for the integration [19], and Ccl19‐EYFP^+^ sorted stromal cells [20] whose expression is almost exclusively confined to CD31^neg^PDPN^+^ cells [60]. After integration of the three datasets, 13 FRC clusters were identified, preserving the major subsets including the TRCs, TBRCs, IFRCs, and MedRCs. Almost all clusters consisted of cells from all three datasets, with consistent annotations within the datasets, except for the Bst1^+^Ccl19^+^ TRC cluster, which only contained cells from Perez‐Shibayama et al. [20]. The restriction of this cluster to the dataset of Perez‐Shibayama et al. [20] might be due to biological reasons, such as differences in sampling timepoints or the use of Ccl19‐EYFP^+^ transgenic mice, or might be due to exclusion during filtering in the other datasets. Despite the limitations in the dataset, we could validate our finding that further differentiated FRCs have enhanced MHC class‐II expression on the protein level.

## 5 Material and Methods

### 5.1 Analysis of Murine Cross‐Tissue Fibroblasts scRNAseq Data

The online available cross‐tissue fibroblasts scRNAseq dataset of Buechler et al. [25] was analyzed using their interactive data portal (FibroXplorer.com). In this paper, we only focused on the steady‐state and perturbed‐state data of the murine fibroblasts. Both UMAPs and violin plots were used to visualize different tissues, clusters, and expression of specific markers. The genes identified in the KEGG pathway for antigen processing and presentation were visualized using a heatmap.

### 5.2 Mice

C57BL/6 WT mice were bred and maintained in the animal facility of the Amsterdam UMC (Amsterdam, the Netherlands). The mice were kept under specific pathogen‐free conditions. All animal experiments were reviewed and approved by the National Committee for Animal Experiments.

### 5.3 Human Lymph Nodes

Human LNs were collected from male donors during liver transplant procedures, performed at the Erasmus MC, Rotterdam, The Netherlands. The LNs were resected along the hepatic artery and portal vein in the porta hepatis from the donor liver. The five donors were 48‐, 58‐, and 81‐year‐old males and 23‐ and 46‐year‐old females. LNs were transported in Belzer University of Wisconsin (UW) cold storage solution (Bridge to Life Ltd) and processed within 72 h after surgery.

### 5.4 Lymph Node Enzymatic Digestion and CD45^neg^ Cell Enrichment

Skin draining LNs from five male C57BL/6 mice (6 weeks old) or six female C57BL/6 mice (6 months old) were harvested and enzymatically digested as previously described [61]. In short, isolated LNs were resuspended in RPMI 1640 medium (Gibco) supplemented with Collagenase P (0.2 mg/mL), Dispase II (0.8 mg/mL), and DNase I (0.1 mg/mL) (all from Sigma‐Aldrich) and incubated for cycles of 15 min at 37°C. The LNs were resuspended, and the supernatant containing LN cells was collected in phosphate‐buffered saline (PBS) supplemented with 5 mM EDTA and 2% fetal calf serum (FCS) to prevent overdigestion and neutralize the digestion enzymes. Fresh digestion medium was added to the LNs, and the steps were repeated until the LN structure was fully lost, with a maximum of five digestion rounds. The whole LN cell suspensions were spun down at 300 g for 5 min at 4°C, resuspended in PBS containing 5 mM EDTA and 2% FCS, and filtered through a 70 µM filter and counted. The whole murine LN cell suspension was enriched for CD45^neg^ (stromal) cells using CD45‐PE antibody (30‐F11, 1:1000, Biolegend) and the EasySep PE Positive Selection Kit II (Stem Cell), following the manufacturer's protocol. The CD45^neg^‐enriched murine LN cell suspensions were used to isolate FRCs for scRNAseq (6‐week‐old mice) or to perform flow cytometry analysis (6‐month‐old mice).

Human LNs were enzymatically digested similarly as described for murine LNs, with differences in the enzyme concentrations: Collagenase P (0.6 mg/mL), Dispase II (2.4 mg/mL), and DNase I (0.3 mg/mL); the time of each digestion cycle: 10 min; the resuspension buffer used: Dulbecco's modified Eagle medium (DMEM) (GIBCO) supplemented with 10% FCS, 2% penicillin/streptomycin/glutamine and 1% insulin/transferrin/selenium (Gibco); and the filter size: 100 µM [33, 61, 62]. The obtained human LN cell suspensions were enriched for CD45^neg^ (stromal) cells by negative selection using MojoSort Human CD45 Nanobeads (BioLegend). The manufacturer's protocol was followed, with the addition of a fluorescently labelled antibody against CD45 (eF450, 1:100, clone HI30, eBioscience) during the CD45 Nanobeads incubation step to enhance the CD45 signal for flow cytometry. The CD45^neg^‐enriched human LN cell suspension was further processed for flow cytometry analysis.

### 5.5 FRC Isolation for Single‐Cell mRNA Sequencing

The CD45^neg^ enriched murine LN cell suspensions were stained for TER119 (BV605, 1:200, clone TER‐119, Biolegend), CD31 (BV786, 1:400, clone 390, Biolegend), GP38/PDPN (AF488, 1:400, clone 8.1.1, Biolegend) and MHC class‐II (AF647, 1:1000, clone M5/114, MO2Ab core facility). Sytox Blue Dye (Invitrogen) was added to the final suspension as a life/death marker. PDPN^+^CD31^neg^ FRCs were FACS single cell/well sorted into two 384‐wells cell capture microplates, containing either MHC class‐II^+^ (fluorescent intensity MHC class‐II AF647> 300) or MHC class‐II^neg^ (fluorescent intensity MHC class‐II AF647< 100) FRCs, using the BD FACSAria Fusion (BD Biosciences) with a 100 µm tip. The fluorescence intensity for MHC class‐II per sorted cell was measured to combine with the RNA sequencing of the same cell. After spinning down and storage at −80°C, the plates were shipped for library generation and single‐cell mRNA sequencing (scRNAseq) by Single Cell Discoveries (Utrecht, the Netherlands), according to the SORT‐Seq method [63].

### 5.6 Analysis of Murine FRC scRNAseq Data

The gene expression count matrices were processed and analyzed using the Seurat package (version 4.1.1) in R (version 4.2.0). After removal of low‐quality cells (<750 nFeatures, <1000 nCounts), the data were normalized via the LogNormalize method and scale factor of 10000 using the “NormalizeData” function. Next, highly variable genes were identified by “FindVariableFeatures”, and the data were scaled and centered using the linear model in the “ScaleData” function. Principal component analysis was performed using “RunPCA”, which was used for dimensionality reduction using t‐distributed stochastic neighbor embedding (t‐SNE) and cell clustering, using “RunTSNE” and “FindClusters”, respectively. The resolution of “FindClusters” was set to 0.4, because the resulting five clusters were consistent with the mapping of known markers. Differentially expressed genes (DEGs) were identified using the “FindAllMarkers” function with the default parameters and used to annotate these five clusters.

### 5.7 Integration with Public Datasets

Two publicly available datasets, from Kapoor et al. [19] and Perez‐Shibayama et al. [20], were selected for integration with our scRNAseq dataset. For the single‐cell mouse FRC dataset of Kapoor et al. [19], the processed data, including the clustering and annotation, are publicly available (E‐MTAB‐10197). This dataset consists of CD45‐depleted skin‐draining LN cells from 5–9 weeks old mice [19]. Only the mesenchymal stromal cells (CD45^neg^CD31^neg^PDPN^+/neg^) were used for the integration. For the single‐cell mouse dataset of Perez‐Shibayama et al. [20], the raw data is publicly available (E‐MTAB‐8906). We preprocessed the data of the Ccl19‐EYFP^+^ sorted stromal cells from the skin‐draining LNs of 8–10 weeks old naive mice, as described above for our scRNAseq dataset. This included dimensionality reduction, cell clustering, and identifying DEGs to annotate the different clusters. We removed two endothelial clusters, expressing *Cd31*, and two clusters highly expressing mitochondrial genes, indicating apoptotic or lysing cells. The other clusters were annotated based on the markers described for the different clusters within the Perez dataset, although we did identify three TBRC clusters compared with the two populations described before [20].

For the integration of the three datasets, we followed version 3 of the integration workflow outlined in the Seurat package v3, previously found here: https://satijalab.org/seurat/archive/v3.2/integration.html.

This integrated dataset was further analyzed using dimensionality reduction, cell clustering, and identifying DEGs. The 13 clusters, which were identified with Seurat's “FindClusters” function at a resolution of 0.5, were annotated based on the DEGs and the previously annotated clusters from the original datasets. Pseudotime analysis was performed using the Monocle3 package (version 1.3.1), with selection of the Cd34^+^Cd26^+^Cd90^+^ SC as the starting point as described before [19].

### 5.8 Flow Cytometry

CD45^neg^ enriched murine and human LN cell suspensions were stained in a 96‐well U‐bottom plate at 4°C for flow cytometry analysis. First, cells were washed with PBS and stained with a fixable viability dye (eF780, 1:2000, Invitrogen) for 10 min at 4°C. Next, cells were washed with PBS containing 2% FCS (referred to as flow cytometry buffer) prior to Fc‐receptor blocking using 5% normal mouse serum or 10% normal human serum in flow cytometry buffer mixed 1:1 with Brilliant Stain Buffer (BSB) (BD Biosciences), for mouse or human samples, respectively. Cells were then incubated with directly labeled antibodies diluted in blocking buffer and BSB. See Table 1 for antibodies and dilutions used. After staining, cells were washed two times with flow cytometry buffer and fixed with 2% paraformaldehyde (PFA) for 15 min at 4°C, and washed two times with flow cytometry buffer. Samples, single stains on beads, as well as Fluorescence Minus One (FMO) controls, were acquired on the BD LSRFortessa X‐20 conventional flow cytometer (BD Biosciences) for mouse samples, and on Aurora 5‐laser spectral flow cytometer (Cytek), for human samples, due to their autofluorescence. Before analysis, the heterogeneous and bright autofluorescence spectra seen in human LN stromal cells were extracted according to our in‐house developed analysis pipeline [64]. Data were analyzed and visualized using FlowJo Software (v10.7, TreeStar) and Graphpad Prism9 software.

**TABLE 1:** Antibody panels used for flow cytometry.

Sample	Target	Fluorochrome	Clone	Dilution	Manufacturer
Mouse	CD45	eF450	30F11	200	Invitrogen
	Ter119	BV605	Ter119	400	Biolegend
	PDPN	AF488	8.1.1	200	Biolegend
	CD31	BV785	390	400	Biolegend
	CD157/Bst1	PE	BP3	600	Biolegend
	CD200 (Ox2)	Pe‐dazzle594	OX90	600	Biolegend
	MHCII	AF647	M5/114	1000	MO2Ab facility
Human	CD45	eF450	HI30	100 (used in bead selection)	eBioscience
	CD235a	eF450	HIR2 (GA‐R2)	10	eBioscience
	CD31	BV605	WM59	100	Biolegend
	CD146	BV785	P1H12	100	Biolegend
	BST1	BV750	SY/11B5	200	BD Optibuild
	CD200	APC	OX104	25	eBioscience
	HLA‐DR	PE	L243	100	Biolegend
	CD21	Pe‐dazzle594	Bu32	50	Biolegend

### 5.9 Immunohistochemistry

Immunohistochemistry was performed on sequential, paraffin‐embedded sections (5 µm) of healthy human LNs. First, sections were deparaffinized, and antigen retrieval was achieved by incubating the sections in Tris‐EDTA buffer (Target Retrieval Solution, high pH, Agilent) at 100°C for 10 min. Next, endogenous peroxidase activity was reduced via treatment of the sections with peroxidase‐blocking solution (Dako REAL, Agilent) for 10 min at room temperature (RT). After washing with PBS, the sections were blocked to prevent nonspecific binding for 30 min at RT with PBS supplemented with 5% bovine serum albumin (BSA) and 10% normal rabbit serum. Then, sections were incubated with primary antibody diluted in 1% BSA in PBS for 60 min at RT. The sequential sections were stained with primary antibodies against CD3 (clone F7.2.38, Agilent), HLA‐DR (clone TAL.1B5, Agilent), CD200 (polyclonal, R&D systems), or PDPN (clone LpMab‐7, Abcam). After three washes, sections were incubated with horseradish peroxidase‐labelled secondary antibodies for 30 min at RT, followed by visualization with the peroxidase substrate diaminobenzidine (DAB+ substrate‐chromogen solution, Dako, Agilent). Before embedding in Entellan (Merck, Sigma), the sections were counterstained with hematoxylin. Stained sections were imaged with the VS200 slide scanner (Olympus) and analyzed with QuPath (v0.2.2).

### 5.10 Statistical Analysis

All data other than the scRNAseq were analyzed using Prism9 software (Graphpad). Differences were analyzed by the Kruskal–Wallis test with Dunn's multiple comparisons test or one‐way ANOVA with a Tukey's multiple comparison test, as indicated in figure legends. In all figures, *p < 0.05; **p < 0.01; ***p < 0.001; ****p < 0.0001.

## Author Contributions

**Janna E. G. Roet**: conceptualization; data curation; formal analysis; investigation; methodology; software; validation; visualization; writing – original draft preparation; writing‐ review and editing. **Catarina Gago da Graça**: conceptualization; investigation; methodology; writing‐ review and editing. **Michael de Kok**: software; writing‐ review and editing. **Daphne Panocha**: investigation. **Tanja Konijn**:: investigation. **Henk P. Roest**: resources. **Luc J.W. van der Laan**: resources. **Lisa G. M. van Baarsen**: Conceptualization; Funding Acquisition; Methodology; Writing‐ Review and Editing. **Charlotte M. de Winde**: Conceptualization; Methodology; Supervision; Writing‐ Review and Editing. **Reina E. Mebius**: Conceptualization; Data Curation; Funding Acquisition; Methodology; Supervision; Writing‐ Review and Editing.

## Ethics Statement

All animal experiments performed were approved by the National Committee for Animal Experiments under CCD AVD1140020173665. Human LNs were obtained from donors during liver transplant procedures performed at the Erasmus MC, Rotterdam, the Netherlands, in accordance with the Medical Ethical Committee (Medisch Ethische Toetsings Commissie; METC) of Erasmus MC (MEC‐2014‐060). All liver transplant recipients gave written informed consent for the use of their donor tissue.

## Conflicts of Interest

The authors declare no conflicts of interest.

## Supporting information




**Supporting Information File 1**: eji70086‐sup‐0001‐SuppMat.pdf

## Data Availability

scRNAseq data have been deposited and are publicly available in the SRA database under the bioproject accession number PRJNA1223898. The additional data that support the findings of this study are available from the corresponding author on reasonable request.
