# Supplementary material for: Enhanced MHC Class‐II Expression in Fibroblastic Reticular Cells Associates with Maturation
Source: Eur J Immunol. 2025 Nov 10;55(11):e70086. doi: 10.1002/eji.70086 (PMC12599485; doi:10.1002/eji.70086)
Supplement: Supplementary file 1 — Supporting Information File 1: eji70086‐sup‐0001‐SuppMat.pdf [file EJI-55-e70086-s001.pdf]

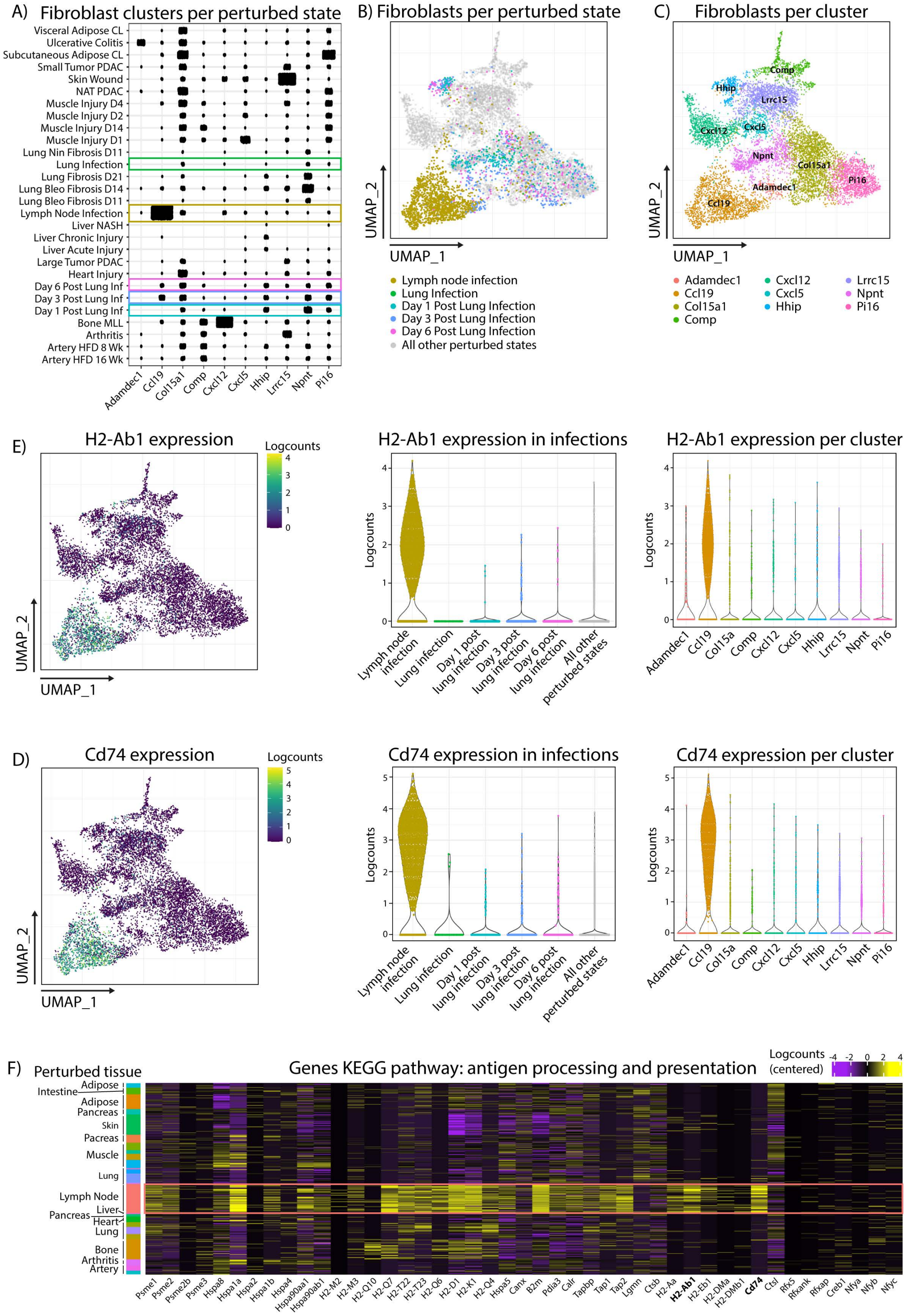

Figure S1. scRNAseq data of murine fibroblasts from various tissues during perturbed states.
A) Distribution of the fibroblast from the 28 different perturbed states over the different clusters. B) UMAP visualization of the fibroblasts from lymph node infection compared to lung infection and other perturbed states. C) UMAP visualization of the fibroblasts per cluster. D) *H2-Ab1* gene expression visualized using UMAP dimensional reduction, violin plot of fibroblasts from lymph node infection vs other perturbed states and violin plot per cluster. E) *Cd74* gene expression visualized using UMAP dimensional reduction, violin plot of fibroblasts from lymph node infection vs other perturbed states and violin plot per cluster. F) Heatmap of the genes present in the KEGG pathway for antigen processing and presentation for the various perturbed tissues. All data is retrieved from Buechler *et al.* (2021) and visualized using FibroXplorer.com.

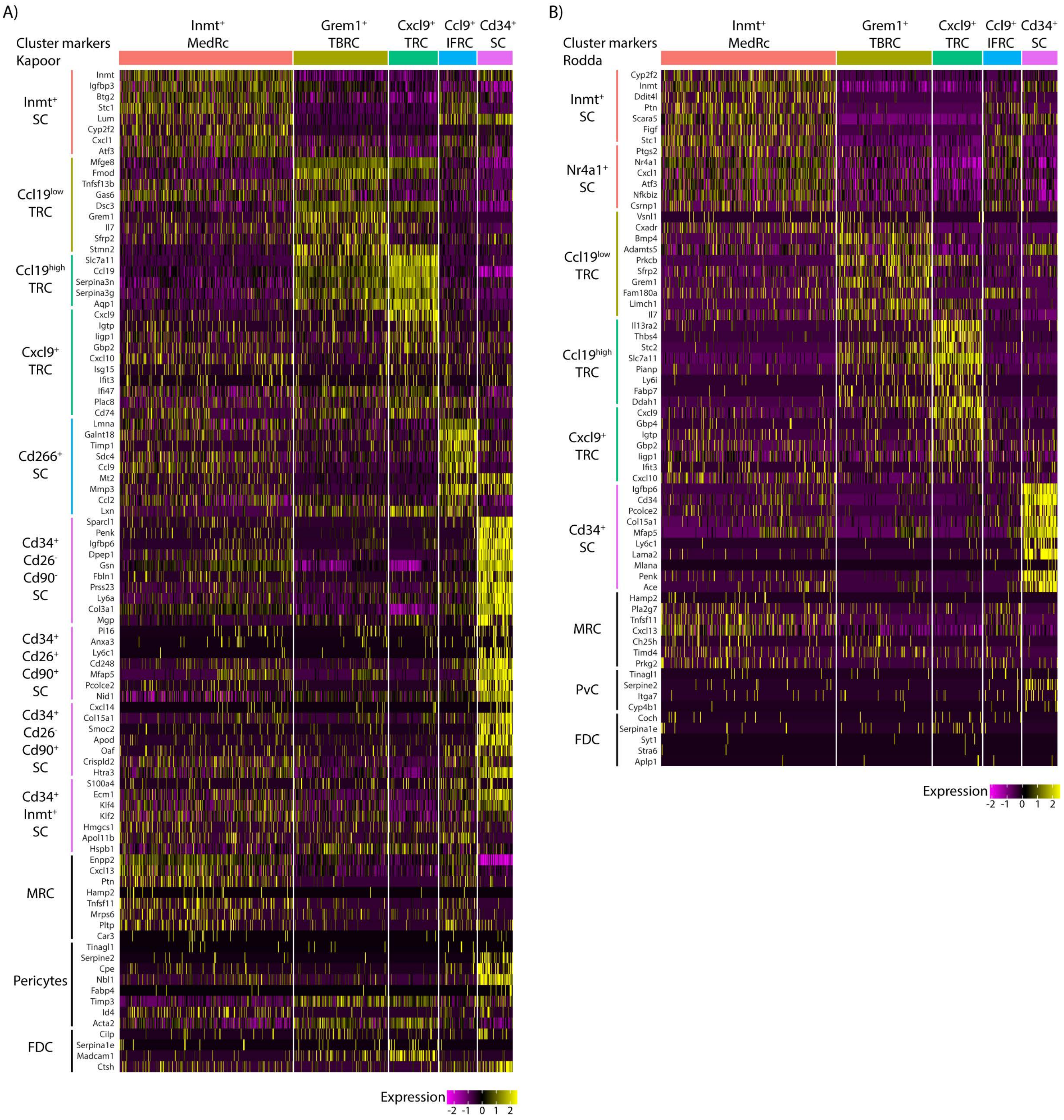

Figure S2. scRNAseq data of murine lymph node FRCs compared to other datasets
A) Heatmap of the murine lymph node FRC scRNAseq data (depicted in Figure 2), visualizing only the genes identified previously by Kapoor *et al*. (2021). Vertical coloured lines visualize the overlap of the gene signatures from the clusters of Kapoor *et al.* (2021) with the five FRC clusters shown in Figure 2. B) Heatmap of murine lymph node FRC scRNAseq data, visualizing only the genes identified previously by Rodda *et al*. (2018). Vertical coloured lines visualize the overlap of the gene signatures from the clusters of Rodda *et al.* (2018) with the five FRC clusters shown in Figure 2.

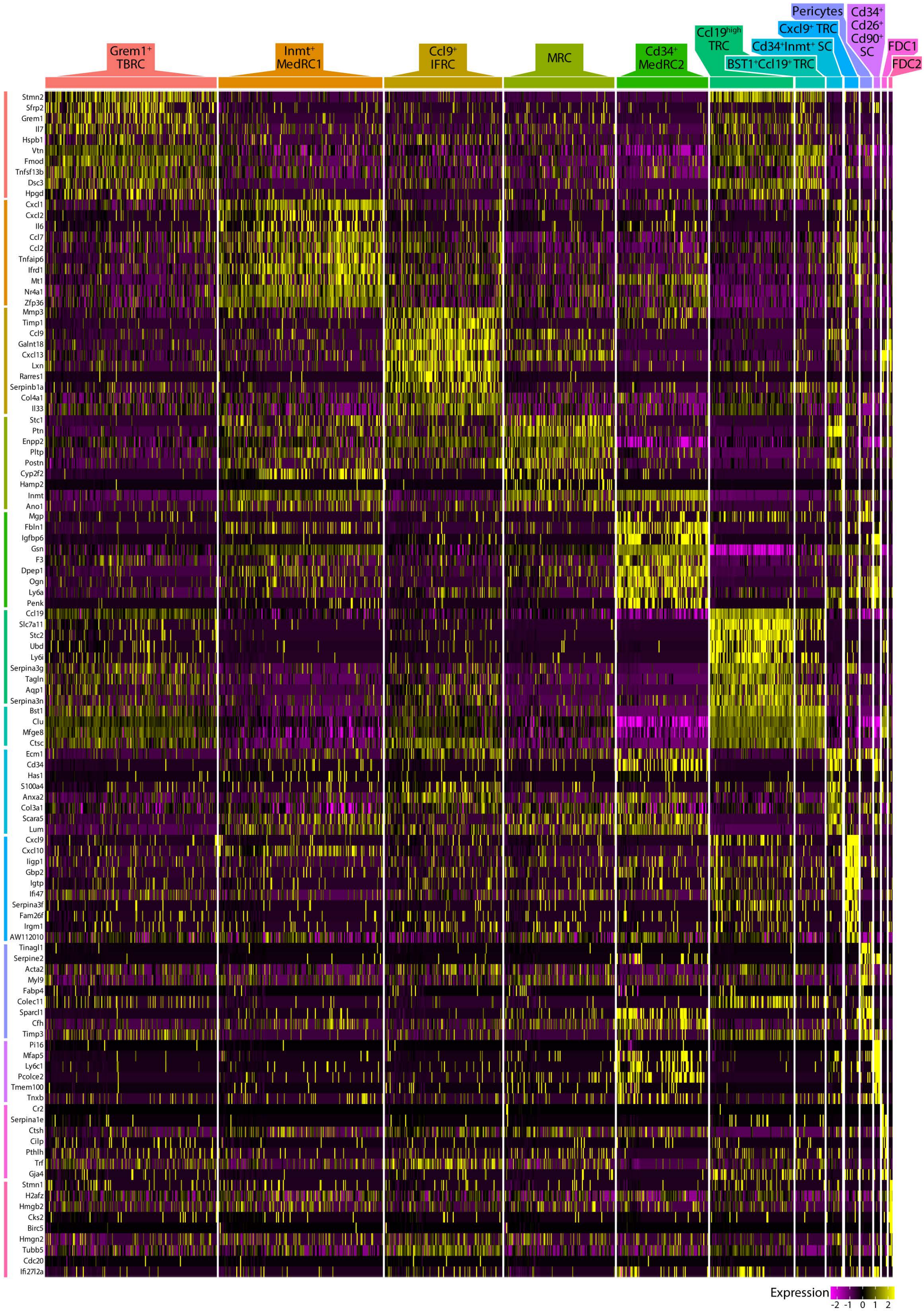

Figure S3. Top 10 differentially expressed genes in the integrated scRNAseq dataset

Heatmap of the integrated murine lymph node FRC scRNAseq dataset of the top 10 significant differentially expressed genes with the highest log fold change per cluster.

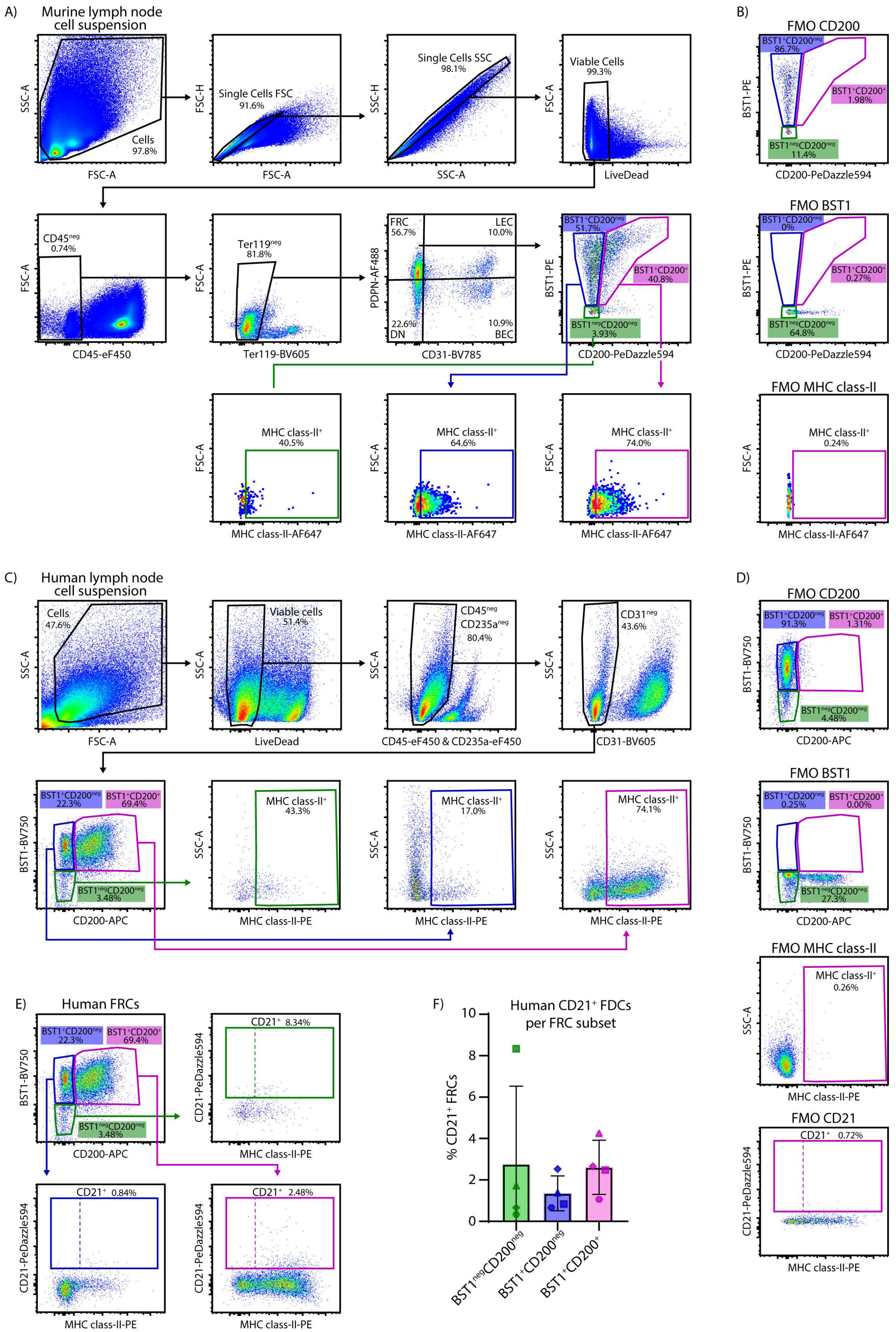

Figure S4. Gating strategy for mouse and human FRCs.

Gating strategies on freshly digested murine (A) and human (C and E) lymph node cell suspensions. Dot plots are shown from one representative sample, including the fluorescent minus one (FMO) controls (B and D) for the main markers. F) Percentage of CD21$^+$ human FRCs, within the three subgroups (n=4). Data is represented as mean ± standard deviation.

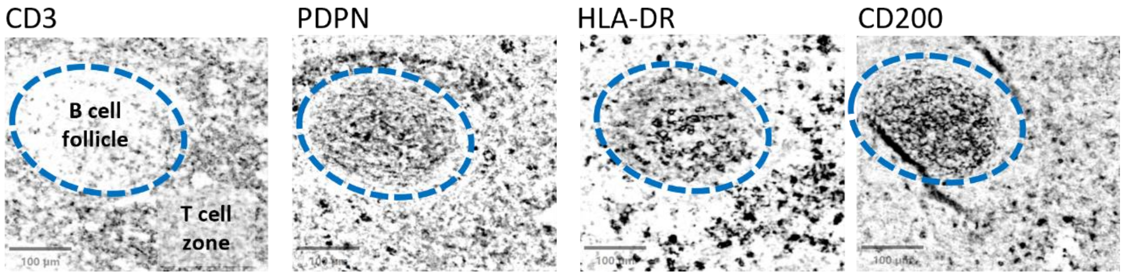

Figure S5. CD200 expression in T cell zone FRCs and B cell follicle FDCs in human lymph node
Immunohistochemistry analysis for CD3, HLA-DR, CD200 and PDPN on sequential human lymph node sections. B cell follicle and T cell zone are indicated by dotted line and scale bars are 100 µm. Representative images of one from five different lymph nodes are shown.
